# Cardioprotective effects of enteral vs. parenteral lactoferrin administration on myocardial ischemia-reperfusion injury in a rat model of stunned myocardium

**DOI:** 10.1186/s40360-022-00619-w

**Published:** 2022-10-14

**Authors:** Keisuke Omiya, Yosuke Nakadate, Takeshi Oguchi, Tamaki Sato, Toru Matsuoka, Masako Abe, Akiko Kawakami, Takashi Matsukawa, Hiroaki Sato

**Affiliations:** 1 Department of Anesthesiology, Faculty of Medicine, University of Yamanashi, 1110 Shimokato, 409-3898 Chuo, Yamanashi Japan; 2 Department of Anesthesia, McGill University Health Centre Glen Site, Royal Victoria Hospital, Montreal, Canada

**Keywords:** Lactoferrin, Rat heart, Ischemia-reperfusion, Langendorff system, Cardioprotection, Protein kinase, Stunned myocardium

## Abstract

**Background:**

Lactoferrin, an iron-binding glycoprotein, is known to have protective effects against intestinal and cerebral ischemia-reperfusion (IR) injuries; however, its cardioprotective effects against the stunned myocardium are unknown. This study aimed to test the hypothesis that lactoferrin has cardioprotective effects against stunned myocardium.

**Methods:**

Using isolated rat hearts (Langendorff system), we determined the effects of lactoferrin administered enterally and by direct cardiac perfusion. Rat hearts were perfused using the Langendorff system, and two experiments were performed. In experiment 1, the hearts were divided into the enteral lactoferrin (E-LF) 7.5 m, 15 m, 30 m, and 60 m groups, where lactoferrin (1000 mg/kg) was administered enterally 7.5, 15, 30, and 60 min, respectively, before perfusion; and a control group, where saline was administered 30 min before perfusion. In experiment 2, hearts were allocated to the perfusate lactoferrin (P-LF) 15 and 100 groups, where 15 mg/L and 100 mg/L lactoferrin were respectively added to the perfusate, and a control group. Each group was perfused for 20 min prior to 15 min of no-flow ischemia with pacing, followed by 20 min of reperfusion. The primary outcome was the maximum left ventricular derivative of pressure development (LV dP/dt max) 15 min after reperfusion. Myocardial phospho-protein kinase B (p-Akt) was assayed using western blotting.

**Results:**

The LV dP/dt max 15 min after reperfusion in the E-LF 15 and 30 m groups was significantly higher than that in the control group. However, the effects disappeared in the E-LF 60 m group. In the second experiment, there were no significant differences in LV dP/dt max. Myocardial p-Akt was not significantly activated in any lactoferrin group.

**Conclusion:**

Cardioprotection was observed 15–30 min after enteral lactoferrin but not by direct cardiac perfusion with lactoferrin. Myocardial p-Akt was not associated with the cardioprotective effect. The cardioprotective effect may be induced by enteral lactoferrin-induced substances.

**Supplementary Information:**

The online version contains supplementary material available at 10.1186/s40360-022-00619-w.

## Background

Ischemia-reperfusion (IR) injury is a complication that arises during cardiac surgery requiring cardiopulmonary bypass (CPB) [1–3]. Myocardial IR injury is induced by activation of the 2Na^+^/Ca^2+^ exchanger and intracellular Ca^2+^ overload [2]. Some anesthetics provide cardioprotective effects against myocardial IR injury via activation of the myocardial phosphatidylinositol 3-kinase/protein kinase B (PI3K/Akt) signaling pathway [4]. Moreover, insulin preconditioning has been shown to preserve cardiac contractility after ischemia via the PI3K/Akt signaling pathway [5, 6] in a stunned myocardium model.

Recently, the Enhanced Recovery After Surgery (ERAS) program has been advocated for to attenuate the stress response to surgery and promote rapid recovery [7, 8]. Although ERAS is relatively new to cardiac surgery, this program has been associated with significantly improved perioperative outcomes [9, 10]. Preoperative nutritional management, such as enteral carbohydrate supplementation, is one of the key factors in perioperative management. While preoperative inflammation leads to worse outcomes [11], in non-cardiac surgery, preoperative enteral carbohydrate supplementation has been shown to attenuate the inflammatory response [12]. However, in cardiac surgery, preoperative enteral carbohydrate supplementation did not attenuate postoperative inflammation [13]. In cardiac surgery, CPB induces severe inflammation via at least three mechanisms: “contact activation” of the immune system following exposure of blood to the foreign surfaces of the CPB circuit, “IR injury” to the brain, heart, lungs, kidney, and liver as a result of aortic cross-clamping, and “endotoxemia” induced by splanchnic hypoperfusion may damage the mucosal barrier, allowing for gut translocation of the endotoxin [14].

Lactoferrin is an 80 kDa iron-binding glycoprotein of the transferrin family and is found in tears, saliva, nasal and bronchial secretions, bile, gastrointestinal fluids, vaginal fluids, semen, urine, and in particularly high levels in breast milk. It exerts antiviral effects by adjusting the immune system [15–17]. Lactoferrin also contributes to the mammalian innate immune system [15] and anti-inflammatory system [18]. Enteral administration of lactoferrin attenuates intestinal and cerebral IR injury in rats [19, 20]. Although lactoferrin activates the PI3K/Akt signaling pathway [21, 22], the relationship between the cardioprotective effects of lactoferrin and the PI3K/Akt signaling pathway in the isolated stunned rat heart remains unknown.

In this study, we tested the hypothesis that enteral lactoferrin administration prior to ischemia provides cardioprotective effects against IR injury in isolated rat hearts. We also investigated the direct effect of perfusate lactoferrin on myocardial IR injury because cardioplegia is frequently employed in cardiac surgery. The primary outcome was the maximum left ventricular pressure derivative (LV dP/dt max) 15 min after reperfusion. We also assessed the role of myocardial phospho-protein kinase B (p-Akt) as a potential mediator.

## Methods

This study was approved by the Ethics Committee on Animal Research of the University of Yamanashi (Protocol number A 2–8, 2020). All animals were euthanized with a diaphragmatic incision under pentobarbital sodium anesthesia.

### Langendorff perfusion system

Male Wistar rats (weighing 300–320 g each) were anesthetized by an intraperitoneal injection of pentobarbital sodium (80 mg/kg body weight). Hearts were excised and quickly immersed in a cold modified Krebs-Henseleit (KH) buffer at 4 °C. The aorta was cannulated, and retrograde arterial perfusion was initiated at a constant pressure of 70 mmHg with a modified KH buffer (NaCl, 118 mmol/L; NaHCO_3_, 25 mmol/L; KCl, 4.7 mmol/L; KH_2_PO_4_, 1.2 mmol/L; MgSO_4_, 1.2 mmol/L; CaCl_2_, 2.0 mmol/L; di-NaEDTA, 0.5 mmol/L; and glucose, 11 mmol/L). The KH buffer was maintained at 37 °C and bubbled with 95% O_2_ and 5% CO_2_. The left ventricle was cannulated with a thin latex balloon via the pulmonary vein and connected to a pressure transducer (DTXPlus DT-12, Argon Critical Care Systems Singapore Pte. Ltd., Singapore) for continuous measurement of the left ventricular (LV) pressure. The balloon was inflated with water to adjust the LV end-diastolic pressure (LVEDP) to 5–10 mmHg. The pulmonary artery was cannulated with a catheter to collect the coronary effluent for the measurement of coronary flow. Hearts with a heart rate (HR) < 200 bpm and frequent arrhythmias at baseline were excluded.

### Experimental protocol

**Fig. 1:**
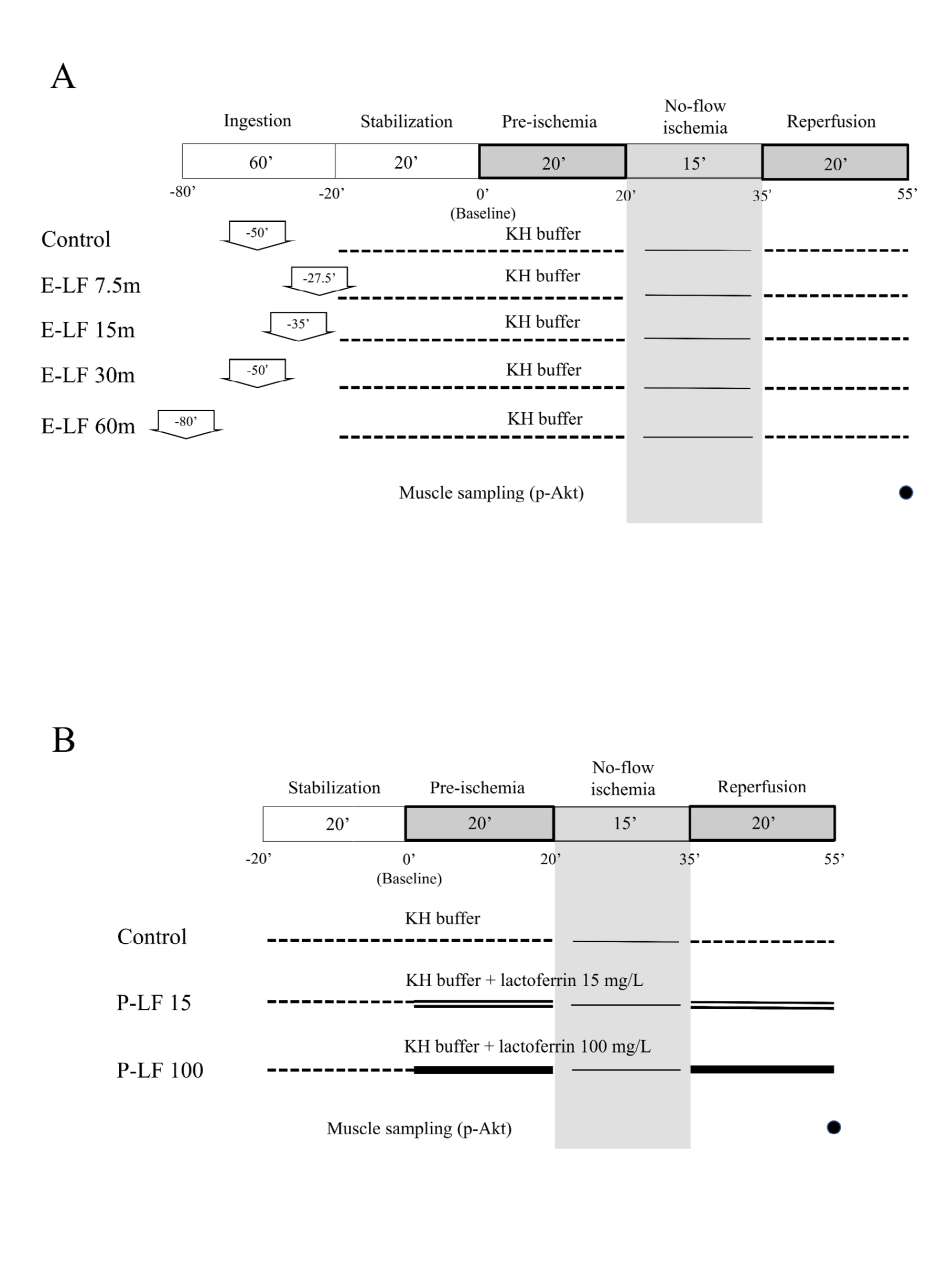
Groups receiving lactoferrin by enteral administration (E-LF) (A) or by perfusate (P-LF) (B). (A) Enteral administration groups. The E-LF 7.5 m, 15 m, 30 m, and 60 m groups received lactoferrin by gavage 7.5, 15, 30, and 60 min, respectively, before intraperitoneal pentobarbital injection. The control group received normal saline by gavage 30 min before intraperitoneal injection. All groups were perfused with KH buffer throughout. (B) Perfusate administration groups. The P-LF 15 and P-LF 100 groups received 15 mg/L and 100 mg/L lactoferrin, respectively, in KH buffer. The control group was perfused with KH buffer throughout. E-LF: enteral lactoferrin, KH: Krebs-Henseleit, p-Akt: phospho-protein kinase B, P-LF: perfusate lactoferrin

*Enteral lactoferrin*: In the first experimental protocol, bovine lactoferrin (protein purity 95%; Fujifilm Wako Pure Chemical Corporation, Osaka, Japan) was used. To assess the effect of enteral lactoferrin administration on myocardial IR injury, rats were randomly divided into five groups (n = 8 per group): E-LF 7.5 m, E-LF 15 m, E-LF 30 m, E-LF 60 m, and control. Lactoferrin (1000 mg/kg in normal saline, volume 4 mL/kg) was administered by gavage using a 2-mL syringe and a 15-gauge ball-tipped feeding needle at 7.5 (E-LF 7.5 m), 15 (E-LF 15 m), 30 (E-LF 30 m) or 60 (E-LF 60 m) min before the intraperitoneal pentobarbital injection. Normal saline (4 mL/kg) was administered to the control group by gavage 30 min before intraperitoneal injection. Following a stabilization period of 20 min for the isolated perfused hearts, baseline hemodynamics were recorded. These groups received KH buffer for 20 min before the ischemic period (which lasted for 15 min) and during 20 min of reperfusion. The hearts were paced at 222 beats/min during no-flow ischemia with an electronic stimulator (SEN-3201, Nihon Kohden Corporation, Tokyo, Japan). The experimental protocol is shown in Fig. 1 (A).

*Perfusate lactoferrin*: In the second experimental protocol, human lactoferrin (protein purity > 90%; Sigma-Aldrich Japan K.K., Tokyo, Japan) was used. To investigate the direct effect of lactoferrin on myocardial IR injury, lactoferrin was added to the perfusate. The hearts were randomly divided into three groups (n = 8 per group): P-LF 15, P-LF 100, and control. The P-LF 15 and P-LF 100 groups received 15 mg/L and 100 mg/L lactoferrin, respectively, in KH buffer for 20 min prior to 15 min no-flow ischemia, and during 20 min of reperfusion. The control group was perfused with KH buffer throughout. Following a stabilization period of 20 min for the isolated perfused hearts, baseline hemodynamics were recorded. During the ischemic period, the hearts were paced at 222 beats/min. The experimental protocol is shown in Fig. 1 (B).

### Measurements

LV dP/dt max (mmHg/s), HR, and LVEDP were continuously recorded for the perfused hearts. Coronary flow (mL/min) was measured by timed collections of the perfusate (baseline, just before ischemia, and after 5, 10, 15, and 20 min of reperfusion) from a catheter inserted into the pulmonary artery.

At the end of reperfusion, each whole heart was promptly frozen in liquid nitrogen and freeze-dried for 6 days before measuring p-Akt/total-Akt in the myocardial muscle by western blot analysis.

### Western blot analysis

The myocardium was suspended in RIPA lysis buffer (Sigma-Aldrich Corp., St. Louis, MO, USA) containing cOmplete™ protease inhibitor tablets (Roche, Basel, Switzerland) and phosphatase inhibitor cocktails 2 and 3 (Sigma-Aldrich Corp.). The samples were then homogenized using the BioMasher II tissue homogenizer (Nippi Inc., Tokyo, Japan). Thereafter, the homogenates were centrifuged for 10 min at 12,000 × g at 4 °C, and the supernatants were collected. The supernatant was diluted in 2 × Laemmli Sample Buffer (Bio-Rad Laboratories, Hercules, CA, USA) containing 5% β-mercaptoethanol and boiled at 95 °C for 5 min. Proteins were separated by 10% sodium dodecyl sulfate-polyacrylamide gel electrophoresis under reducing conditions and then transferred to polyvinylidene difluoride membranes (Bio-Rad Laboratories). After blocking with 5% bovine serum albumin in 0.1% Tween-20 Tris-buffered saline, membranes were incubated with the primary antibodies in 5% bovine serum albumin in 0.1% Tween-20 Tris-buffered saline at 4 °C overnight. The primary antibodies were rabbit anti-Akt and phospho-Akt (Ser473) antibodies (9272, 9271, Cell Signaling Technology, Inc., Danvers, MA, USA), diluted 1:1,000. The membranes were then incubated with the secondary antibody for 1 h. The secondary antibody was HRP conjugated anti-rabbit immunoglobulin G (7074, Cell Signaling Technology, Inc.), diluted 1:1,000. The bands were revealed using an enhanced chemiluminescence detection kit (GE Healthcare Japan Corporation, Tokyo, Japan).

### Statistical analysis

Data are presented as mean ± standard deviation. Comparisons in hemodynamics were analyzed using two-way analysis of variance (ANOVA). Intergroup differences were followed by Dunnett’s test. Comparisons for baseline measurements and p-Akt/total-Akt ratios were analyzed with one-way ANOVA followed by Dunnett’s test. Multiplicity adjusted P-value analysis was performed. Two-sided P-values < 0.05 were considered statistically significant. All statistical analyses were performed using GraphPad Prism version 8 for Windows (GraphPad Software, San Diego, CA, USA). The sample size calculation was based on the expected difference between groups in the LV dP/dt max 15 min after reperfusion, using Power and Sample Size Calculation version 3.1.6 (available at http://biostat.mc.vanderbilt.edu/wiki/Main/PowerSampleSize). The results of our pilot study showed 1000 ± 500 mmHg/s and 2000 ± 500 mmHg/s in the control and E-LF 30 m groups, respectively, 15 min after reperfusion. In order to achieve a power level of 80%, with an alpha error of 5%, at least seven subjects were required in each group.

## Results

There were no significant differences in baseline values among the groups in either experiment (Table 1).

**Table 1:** Baseline measurements

**A. Enteral administration groups**					
	Control	E-LF 7.5m	E-LF 15m	E-LF 30m	E-LF 60m
Number (n)	8	8	8	8	8
LV dP/dt max (mmHg/s)	2500 ± 213	2556 ± 253	2608 ± 271	2669 ± 321	2391 ± 147
Heart rate (bpm)	236 ± 25	234 ± 24	226 ± 21	244 ± 18	255 ± 42
Coronary flow (mL/min)	14.1 ± 1.3	13.3 ± 0.7	14.4 ± 0.9	14.0 ± 1.7	13.0 ± 1.5
**B. Parenteral administration groups**					
	Control	P-LF 15	P-LF 100		
Number (n)	8	8	8		
LV dP/dt max (mmHg/s)	2583 ± 374	2546 ± 420	2569 ± 289		
Heart rate (bpm)	270 ± 42	256 ± 52	246 ± 29		
Coronary flow (mL/min)	15.7 ± 1.8	14.4 ± 0.9	14.7 ± 1.7		

Data are presented as mean ± standard deviation.

E-LF: enteral lactoferrin, LV dP/dt max: maximum left ventricular derivative of pressure development, P-LF: perfusate lactoferrin.

**Fig. 2:**
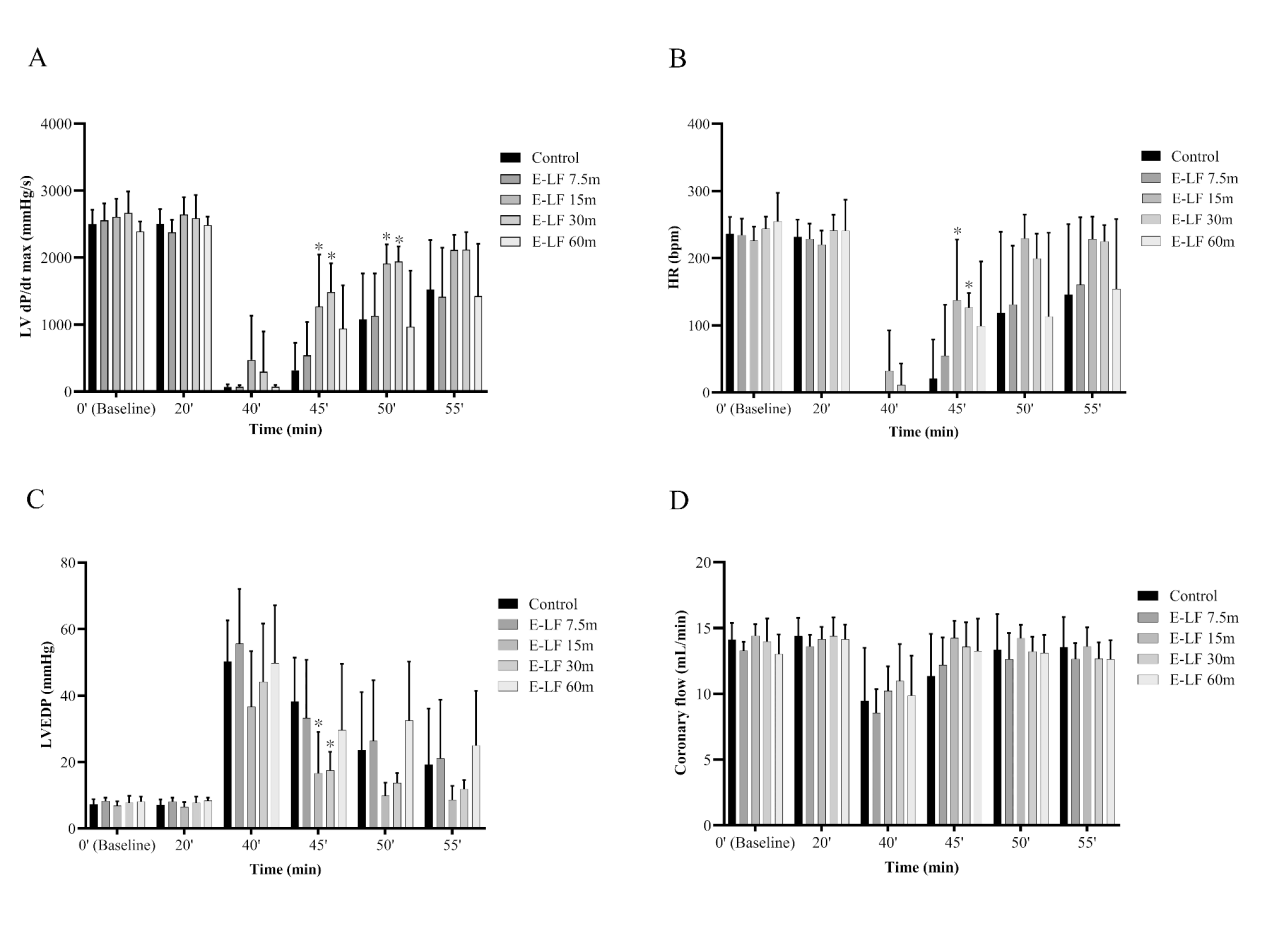
Cardiodynamic changes over time, before and after ischemia, in the enteral administration groups. The following variables were measured for each group (n = 8): (A) LV dP/dt max (mmHg/s), (B) HR (bpm), (C) LVEDP (mmHg), and (D) coronary flow (mL/min). Bars represent means and error bars represent standard deviation. ^*^ P < 0.05 vs. control. E-LF: enteral lactoferrin, HR: heart rate, LV dP/dt max: maximum left ventricular derivative of pressure development, LVEDP: left ventricular end-diastolic pressure

**Fig. 3:**
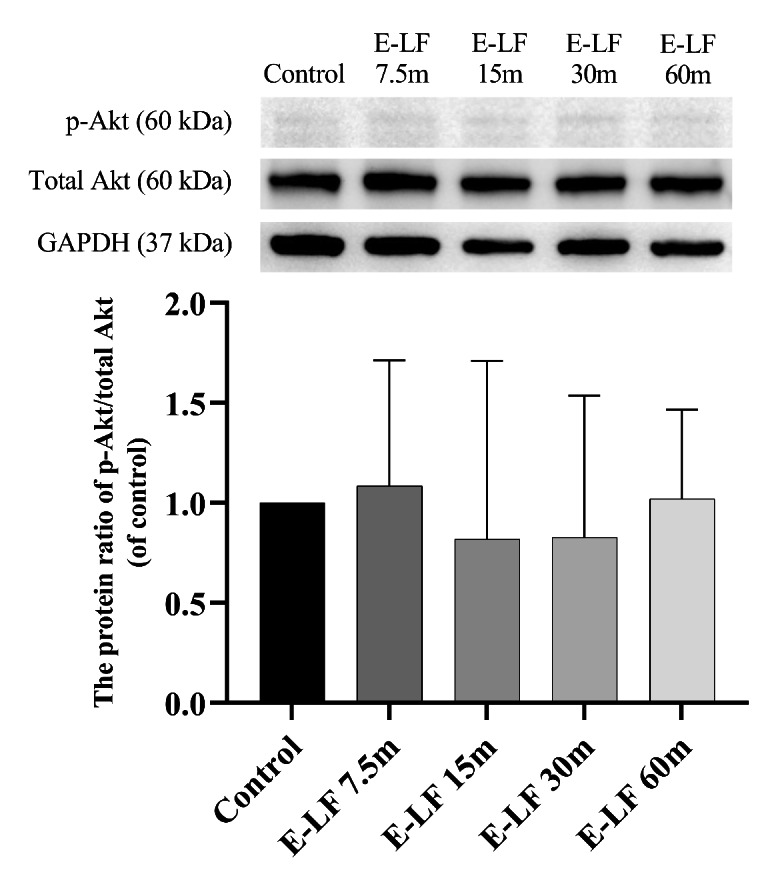
p-Akt protein ratio over total Akt against control, 20 min after reperfusion, enteral administration groups. Bars represent mean and error bars represent standard deviation (n = 3 hearts from eight rats in each group). The mean of p-Akt over total Akt in the control group was normalized to 1. E-LF: enteral lactoferrin, GAPDH: glyceraldehyde 3-phosphate dehydrogenase, p-Akt: phospho-protein kinase B

In the experiment with enteral lactoferrin, the LV dP/dt max of the E-LF 15 m and E-LF 30 m groups at 45 min (mean difference versus control, 957.5 mmHg/s; 95% confidential interval (CI), 73.9–1841.1; P = 0.033, and mean difference versus control, 1168.8 mmHg/s; 95% CI, 591.8–1745.7; P < 0.001, respectively) and 50 min (mean difference versus control, 836.3 mmHg/s; 95% CI, 70.8–1601.7; P = 0.033, and mean difference versus control, 867.5 mmHg/s; 95% CI, 109.6–1625.4; P = 0.026, respectively) (10 and 15 min after reperfusion) was significantly higher than that of the control group (Fig. 2 A). The E-LF 15 m and E-LF 30 m groups at 45 min (10 min after reperfusion) also showed significantly higher HR (mean difference versus control, 116.8 bpm; 95% CI, 10.1–223.4; P = 0.031, and mean difference versus control, 105.8 bpm; 95% CI, 40.7–170.8; P = 0.003, respectively) and lower LVEDP (mean difference versus control, -21.6 mmHg; 95% CI, -39.3 – -4.3; P = 0.015, and mean difference versus control, -20.8 mmHg; 95% CI, -35.5 – -6.0; P = 0.008, respectively) than the control group (Fig. 2B C). However, there were no significant differences in coronary flow compared with the control group (Fig. 2D). Western blotting showed that myocardial p-Akt was not significantly activated in the enteral lactoferrin groups compared with the control group (Fig. 3). The original source data sets in the enteral administration groups are available in Additional File 1. Full-length blots/gels are presented in Additional File 2.

**Fig. 4:**
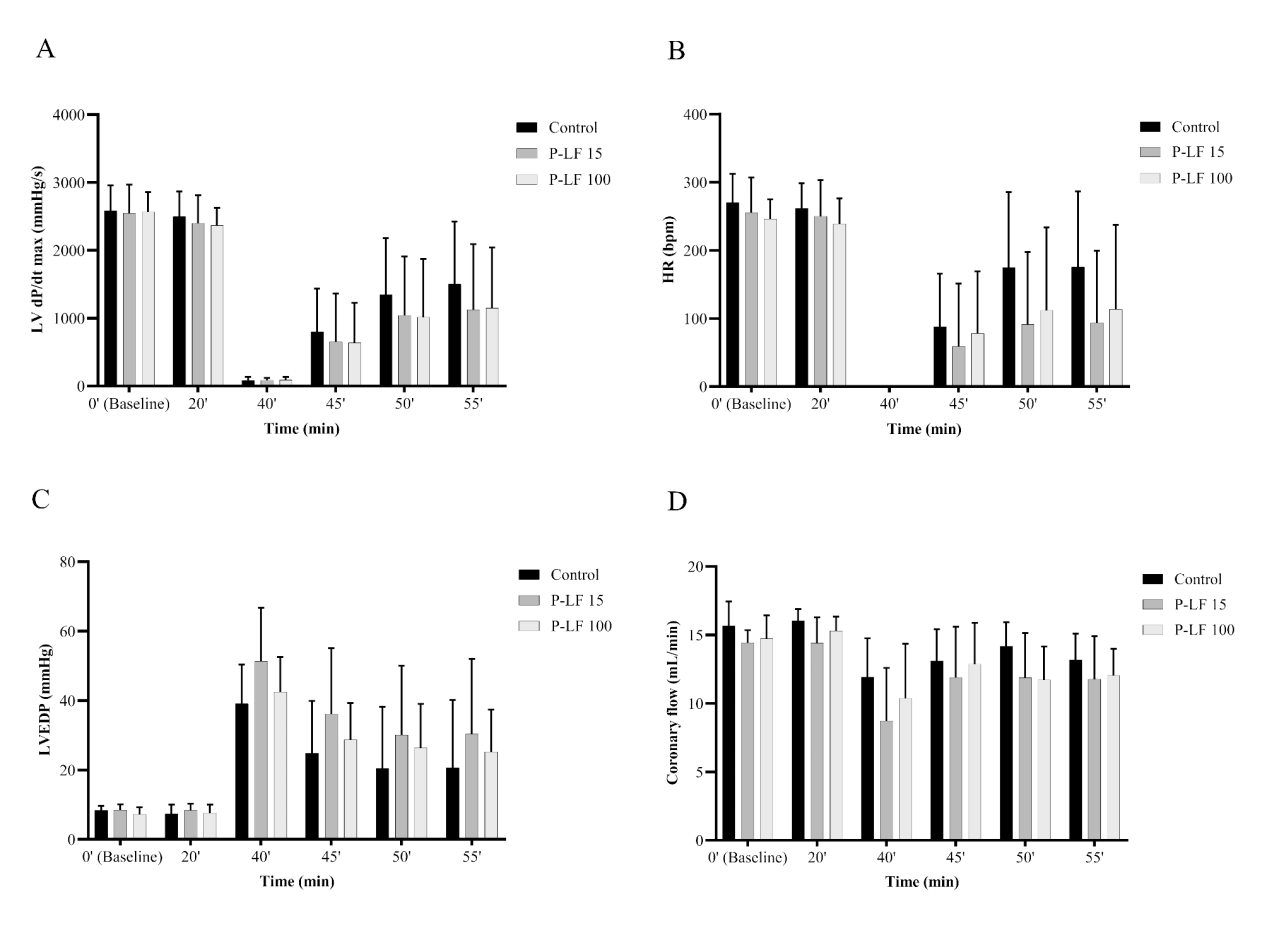
Cardiodynamic changes over time, before and after ischemia, in the perfusate administration groups. The following variables were measured for each group (n = 8): (A) LV dP/dt max (mmHg/s), (B) HR (bpm), (C) LVEDP (mmHg), and (D) coronary flow (mL/min). Bars represent mean and error bars represent standard deviation. HR: heart rate, LV dP/dt max: maximum left ventricular derivative of pressure development, LVEDP: left ventricular end-diastolic pressure, P-LF: perfusate lactoferrin

**Fig. 5:**
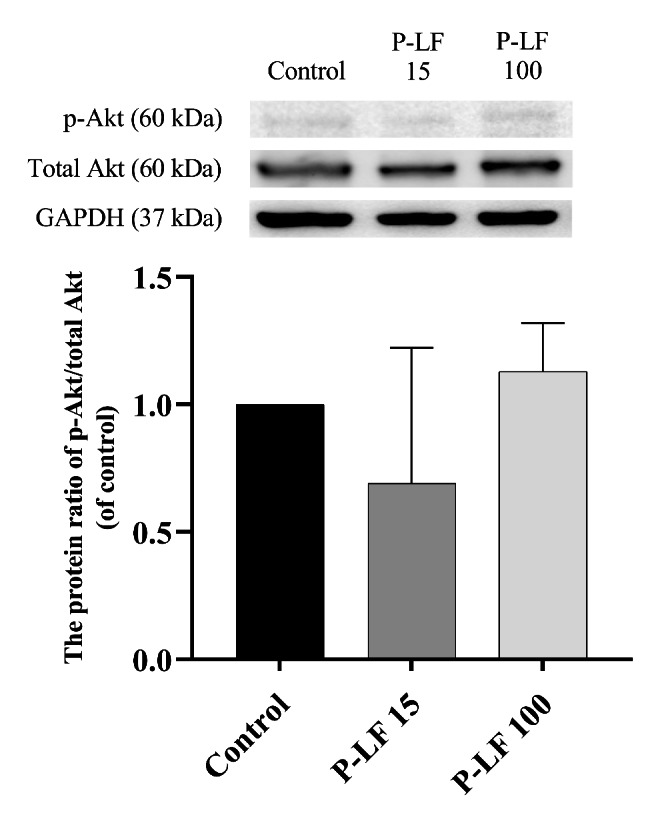
p-Akt protein ratio over total Akt against control, 20 min after reperfusion, perfusate administration groups. Bars represent mean and error bars represent standard deviation (n = 3 hearts from eight rats in each group). The mean of p-Akt over total Akt in the control group was normalized to 1. GAPDH: glyceraldehyde 3-phosphate dehydrogenase, p-Akt: phospho-protein kinase B, P-LF: perfusate lactoferrin

In the experiment with perfusate lactoferrin, no significant differences were found between the lactoferrin and control groups in any hemodynamic parameters (Fig. 4). Western blotting showed that myocardial p-Akt was not significantly activated in the P-LF 15 or P-LF 100 groups compared with the control group (Fig. 5). The original source data sets in the parenteral administration groups are available in Additional File 3. Full-length blots/gels are presented in Additional File 2.

## Discussion

In the present study, enteral lactoferrin showed cardioprotective effects 15 and 30 min after ingestion. The assumptions of our power analysis were almost met (LV dP/dt max 15 min after reperfusion, control: 959 ± 648 mmHg/s, E-LF 30 m: 1944 ± 222 mmHg/s). Perfusing isolated hearts with lactoferrin did not show cardioprotective effects. Compared to the controls, no significant activation of p-Akt was observed in any group receiving lactoferrin. These results suggest that the lactoferrin-induced myocardial protection is an indirect effect and that the PI3K/Akt signaling pathway was not involved in the protective mechanism of lactoferrin.

In this study, a rat model of stunned myocardium was used to mimic the context of CPB. The use of specific pharmacological agents for myocardial preconditioning can mitigate the adverse effects of CPB or ischemia [6]. Pharmacological options include preemptive administration of volatile anesthetics, nicorandil, and insulin [4–6, 23] to protect cardiomyocytes by exerting anti-inflammatory effects. However, these agents are mainly intraoperative and dosage-limited. Therefore, preoperative nutritional treatment can be important for anti-inflammatory therapy, as emphasized in the ERAS program.

We showed a narrow window of timing in which enteral lactoferrin demonstrated utility at 15–30 min after ingestion but not at 60 min. The dose of enteral lactoferrin used in our study was in accordance with previous reports by Ono et al. [24], Cerven et al. [25], and Takeuchi et al. [26]. The 15–30 min time interval after lactoferrin administration corresponds to the time required for the maximum concentration of lactoferrin to appear in mesenteric fat tissue after gavage administration [24]. While the equivalent time in human is unknown, lactoferrin in the stomach was nearly completely emptied into the intestine in 30 min in healthy volunteers [27]. This suggests the effective timing of enteral lactoferrin administration for CPB related ischemic reperfusion cardiac injury is short. Further clinical studies are needed including the administration via a gastric tube after general anesthesia induction.

Conversely, in the second experiment, perfusate lactoferrin did not yield cardioprotective effects, despite administering concentrations 75 and 500 times higher than the normal plasma concentration (approximately 0.2 µg/mL) [15]. Mladenka et al. [28], using a rat model of catecholamine cardiotoxicity, reported that when lactoferrin was administered intravenously, the stroke volume increased due to the inhibition of increasing peripheral resistance. It is unknown whether lactoferrin affects the myocardium directly; however, lactoferrin can dilate blood vessels. Considering our finding that perfusate lactoferrin had no cardioprotective effects and the reports that enteral lactoferrin is not transported into the blood [29] but rather to the mesenteric fat tissue [24], lactoferrin itself may not have cardioprotective effects. Instead, substances induced or activated after lactoferrin reaches the mesenteric fat tissue might allow it to provide cardioprotective effects.

The cardioprotective effects of volatile anesthetics and insulin are associated with activation of the PI3K/Akt signaling pathway [4–6]. In immature hypoxic-ischemic rat brains, lactoferrin supplementation through lactation decreased brain tumor necrosis factor α and interleukin 6 gene transcription via p-Akt activation [20]. In C57BL/6J mouse vessels, after unilateral hindlimb surgery, lactoferrin also promoted vascular endothelial cell function via the Src/Akt/endothelial nitric oxide synthase-dependent pathway on angiogenesis, thereby contributing to revascularization after ischemia [30]. However, in our experiments, the cardioprotective effects of lactoferrin were not associated with the PI3K/Akt signaling pathway, because the p-Akt in the heart of rats from the lactoferrin groups was not activated, regardless of the route of administration, as shown by western blotting. These findings also support the hypothesis that the protective effects against IR injury may not be induced by lactoferrin itself, but by substances induced or activated by enteral lactoferrin.

Glucagon-like peptide-1 (GLP-1) and adipocytokines may be substances induced or activated by enteral lactoferrin, although there is currently no evidence to support this hypothesis. GLP-1, a hormone secreted from intestinal endocrine L cells, has cardioprotective effects via the cyclic adenosine monophosphate-protein kinase A and protein kinase C signaling pathways [31–33]. Maekawa et al. [34] reported that lactoferrin administered to rats by intraperitoneal injection increased GLP-1 in plasma. Adipocytokines such as omentin, apelin, and adiponectin could also be substances induced or activated by enteral lactoferrin, considering that enteral lactoferrin is transported to the mesenteric fat tissue [24] and is associated with lipid metabolism [35, 36]. Ikoma-Seki et al. [37] reported that lactoferrin induced lipolysis via the cyclic adenosine monophosphate-protein kinase A signaling pathway in rat adipocytes isolated from mesenteric fat. Adipocytokines, which are hormones secreted from fat tissues, are involved in the innate immune mechanism [38] and have anti-inflammatory and cardioprotective effects [39]. In particular, adiponectin can suppress inflammation and attenuate myocardial inflammation and injury because it activates the cyclic adenosine monophosphate-protein kinase A signaling pathway [40] and inhibits the toll-like receptor 4 signaling pathway [41].

We acknowledge some limitations of this study. Firstly, the type of lactoferrin employed in the enteral and perfusate groups differed because of economic cost. However, bovine lactoferrin and human lactoferrin possess high sequence homology and identical functions [42]. Secondly, we did not measure other signaling pathways that may also be involved in the investigated process; therefore, further studies are needed to clarify the mechanism underlying lactoferrin’s cardioprotective effects. Thirdly, we did not measure the concentration of plasma lactoferrin in rats that were administered enteral lactoferrin. However, enteral lactoferrin would not be detected in plasma [29]. And fourthly, in the perfusate administration study, a different dose and duration of perfusate lactoferrin may show different results. Further studies are therefore needed to address these gaps.

## Conclusion

Enteral lactoferrin administration protected cardiac contractility in isolated stunned rat hearts, but no beneficial effects were observed for perfusate administration. The PI3K/Akt signaling pathway was not involved in the protective mechanism of lactoferrin. The present study suggests that the cardioprotective effect may be induced by enteral lactoferrin-induced substances.

## Electronic supplementary material

Below is the link to the electronic supplementary material.

Supplementary Material 1

Supplementary Material 2

Supplementary Material 3

## Data Availability

All data generated or analyzed during this study are included in this article and its additional supplementary information files.

## List of abbreviations

ANOVA: Analysis of variance
CPB: Cardiopulmonary bypass
E-LF: Enteral lactoferrin
ERAS: Enhanced Recovery After Surgery
GLP-1: Glucagon-like peptide-1
HR: Heart rate
IR: Ischemia-reperfusion
LV: left ventricular
LV dP/dt max: Maximum left ventricular derivative of pressure development
LVEDP: left ventricular end-diastolic pressure
p-Akt: Phospho-protein kinase B
PI3K/Akt: Phosphatidylinositol 3-kinase/protein kinase B
P-LF: Perfusate lactoferrin
