# Supplementary material for: Cardioprotective effects of enteral vs. parenteral lactoferrin administration on myocardial ischemia-reperfusion injury in a rat model of stunned myocardium
Source: BMC Pharmacol Toxicol. 2022 Oct 14;23:78. doi: 10.1186/s40360-022-00619-w (PMC9563476; doi:10.1186/s40360-022-00619-w)
Supplement: Supplementary file 2 — Supplementary Material 2 [file 40360_2022_619_MOESM2_ESM.pptx]

## Slide 1
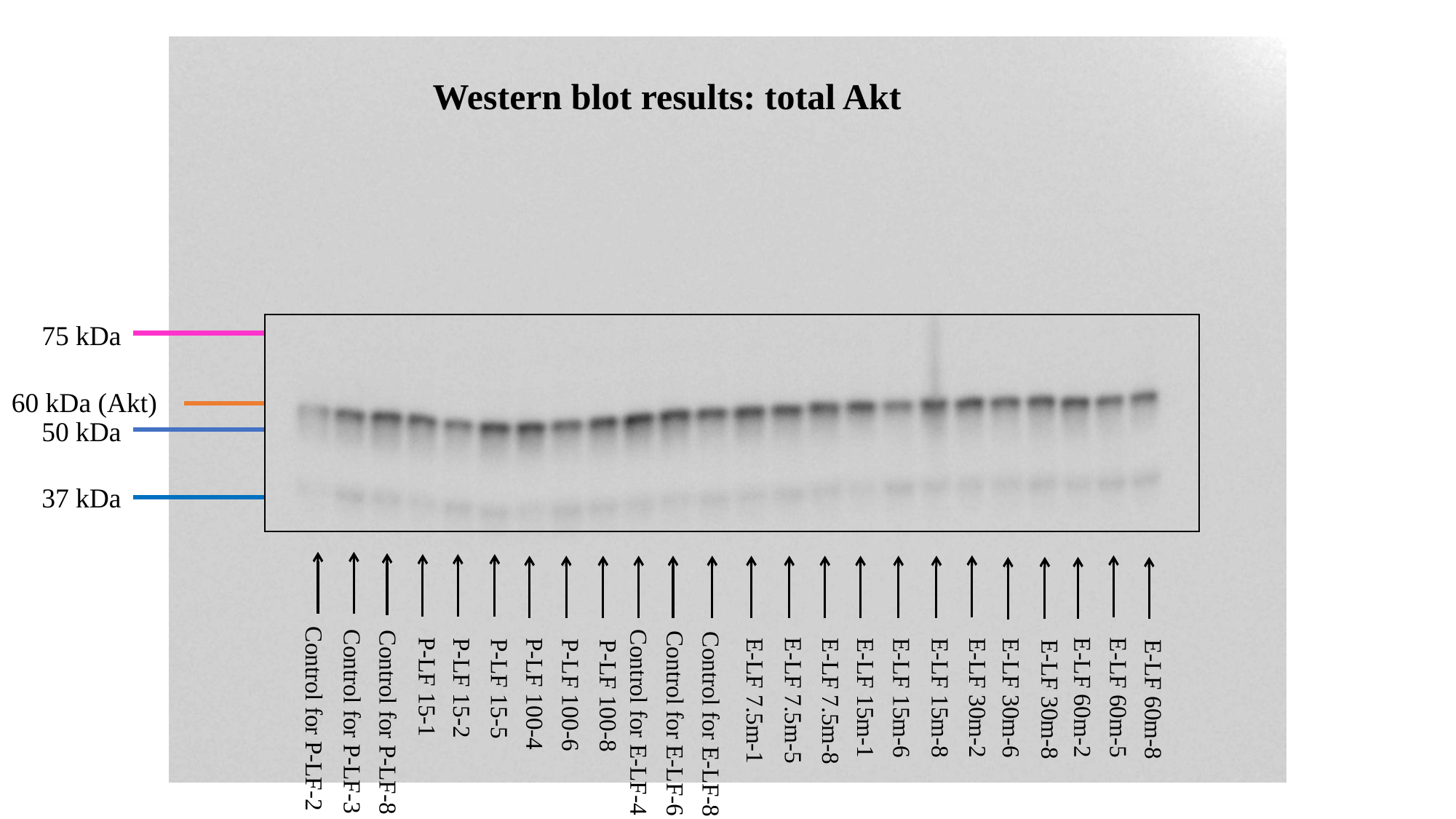

Western blot results: total Akt
75 kDa
60 kDa (Akt)
50 kDa
37 kDa
P-LF 15-1
E-LF 60m-2
E-LF 60m-5
E-LF 7.5m-5
P-LF 100-4
E-LF 30m-6
E-LF 15m-6
E-LF 15m-8
E-LF 30m-2
E-LF 15m-1
P-LF 15-2
E-LF 7.5m-1
E-LF 7.5m-8
P-LF 15-5
P-LF 100-6
E-LF 60m-8
E-LF 30m-8
P-LF 100-8
Control for P-LF-3
Control for P-LF-2
Control for P-LF-8
Control for E-LF-4
Control for E-LF-6
Control for E-LF-8

## Slide 2
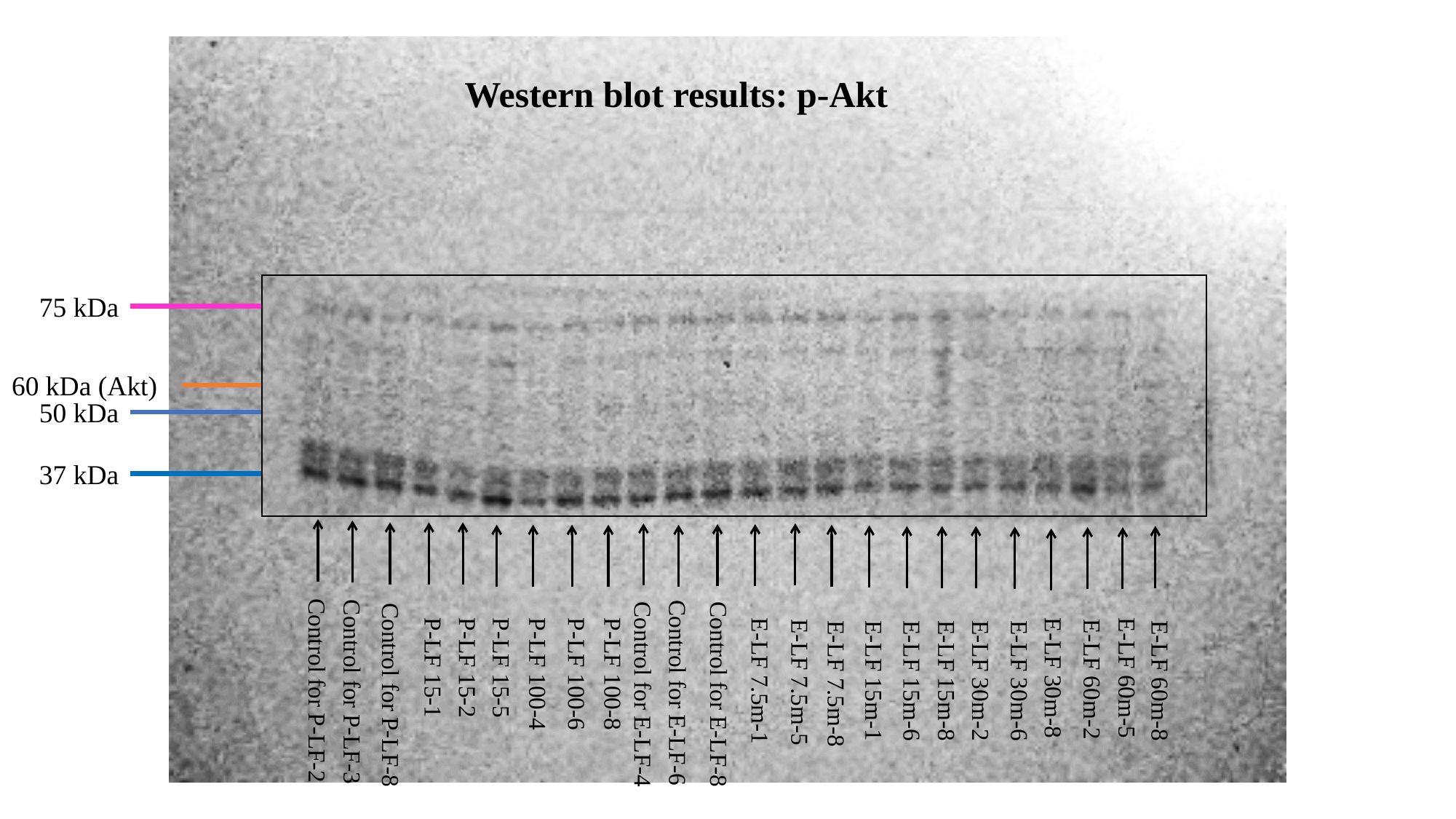

Western blot results: p-Akt
75 kDa
60 kDa (Akt)
50 kDa
37 kDa
E-LF 60m-5
P-LF 100-8
E-LF 7.5m-1
E-LF 30m-8
P-LF 100-4
P-LF 100-6
P-LF 15-1
P-LF 15-2
P-LF 15-5
E-LF 60m-2
E-LF 7.5m-5
E-LF 60m-8
E-LF 30m-6
E-LF 30m-2
E-LF 7.5m-8
E-LF 15m-1
E-LF 15m-6
E-LF 15m-8
Control for P-LF-2
Control for P-LF-3
Control for E-LF-6
Control for E-LF-4
Control for E-LF-8
Control for P-LF-8

## Slide 3
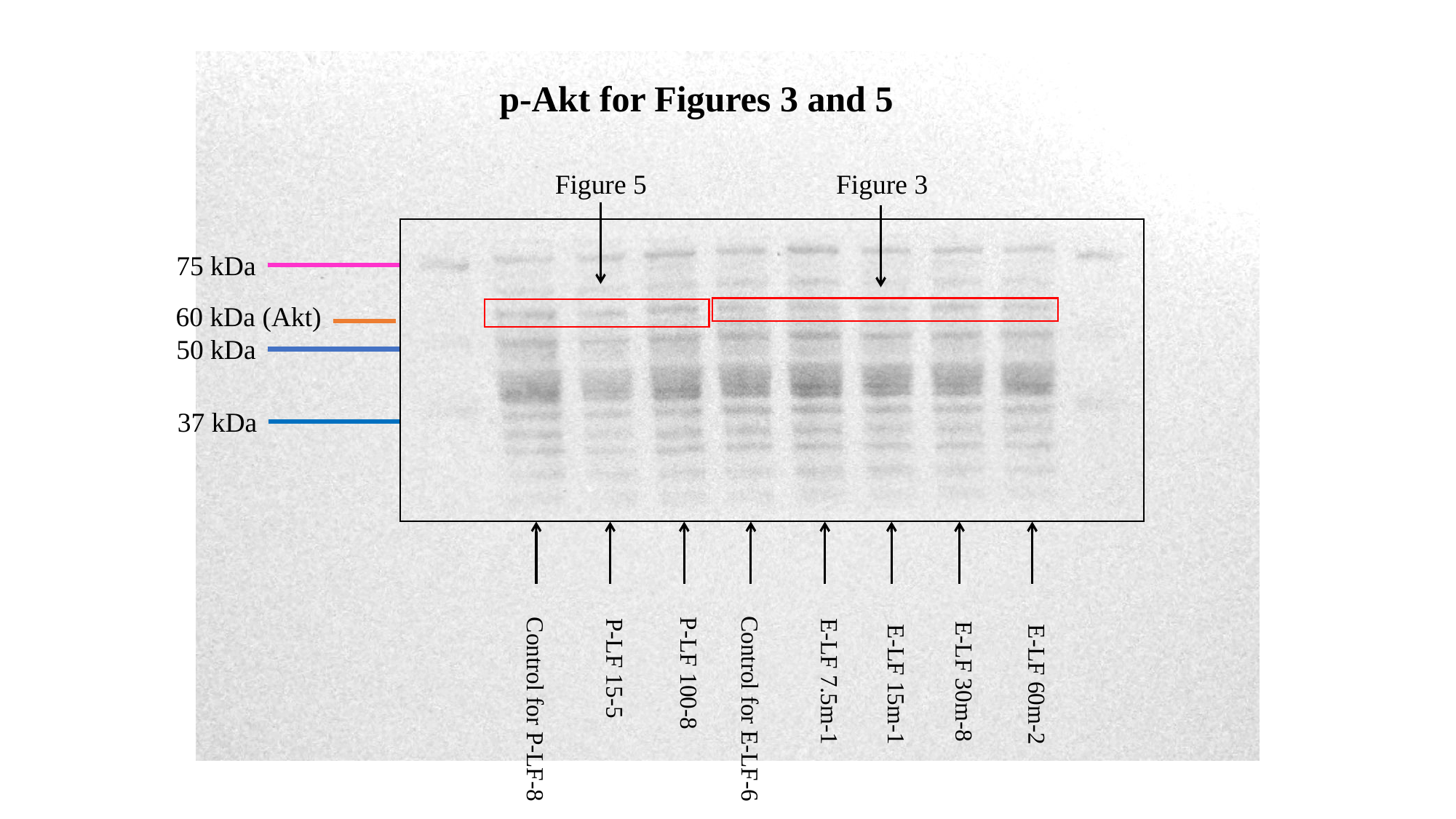

p-Akt for Figures 3 and 5
Figure 5
Figure 3
75 kDa
60 kDa (Akt)
50 kDa
37 kDa
P-LF 100-8
P-LF 15-5
E-LF 7.5m-1
E-LF 30m-8
E-LF 15m-1
E-LF 60m-2
Control for E-LF-6
Control for P-LF-8

## Slide 4
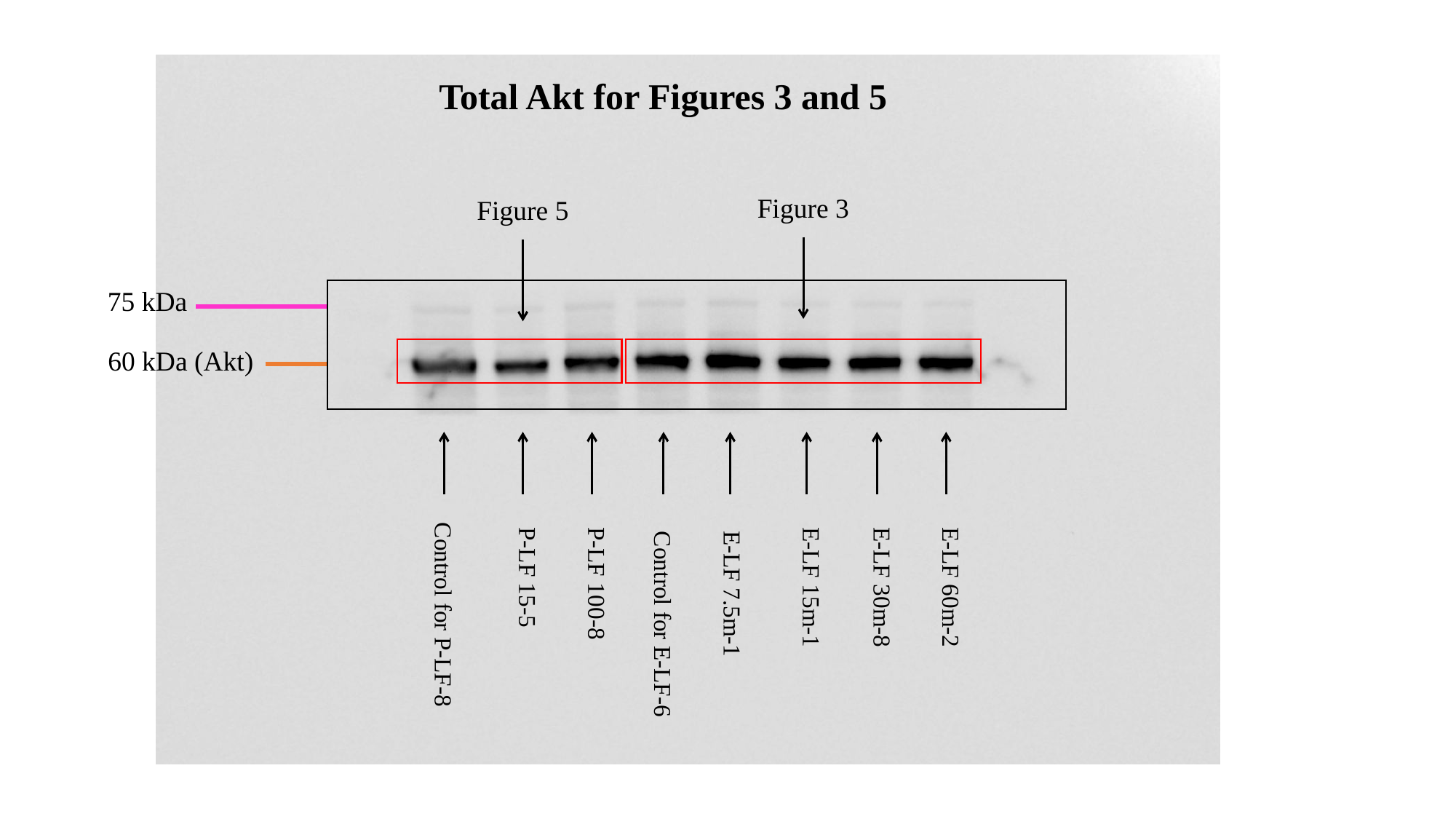

Total Akt for Figures 3 and 5
Figure 3
Figure 5
75 kDa
60 kDa (Akt)
P-LF 15-5
P-LF 100-8
E-LF 15m-1
E-LF 30m-8
E-LF 60m-2
E-LF 7.5m-1
Control for P-LF-8
Control for E-LF-6

## Slide 5
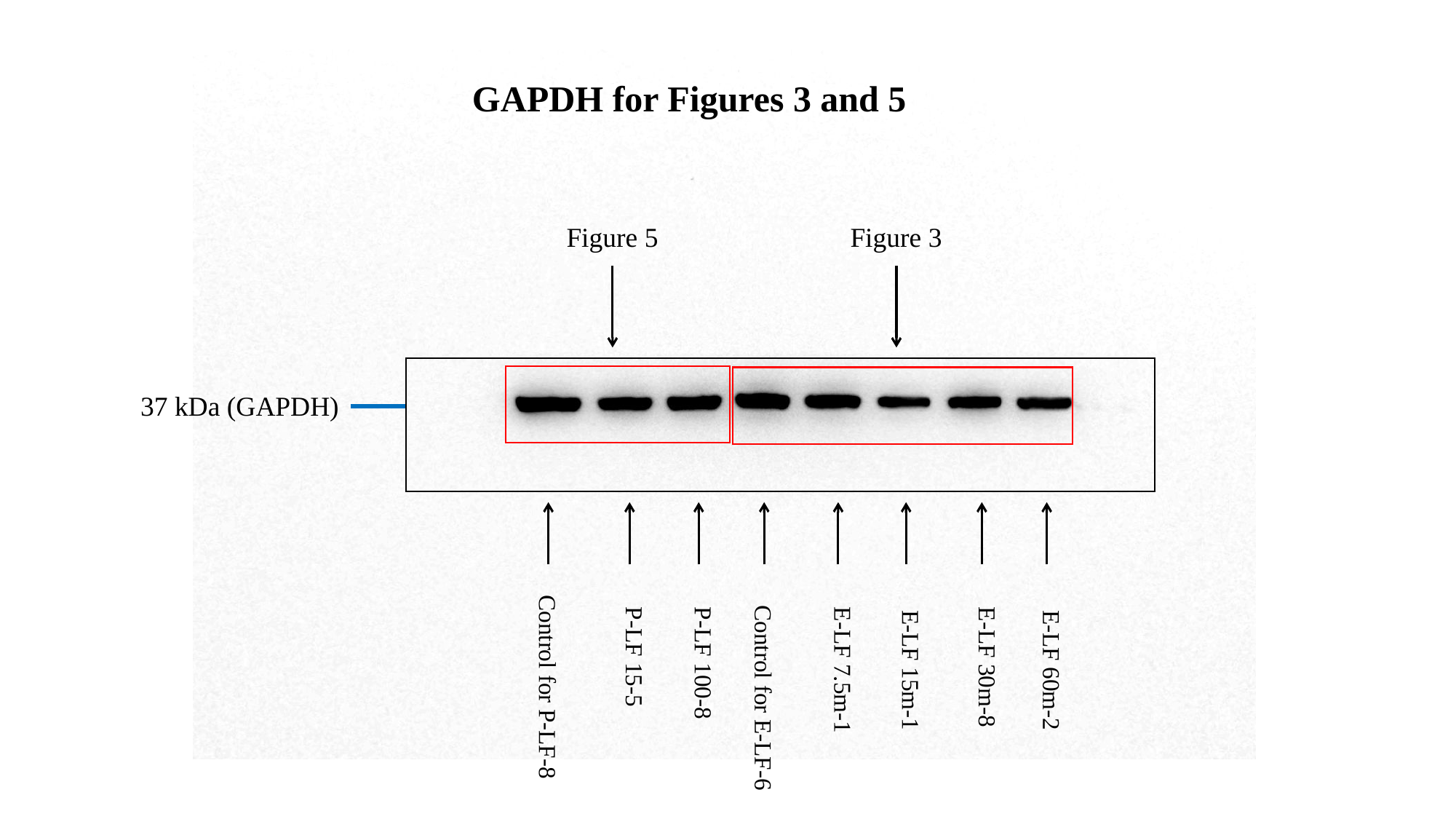

GAPDH for Figures 3 and 5
Figure 5
Figure 3
37 kDa (GAPDH)
P-LF 15-5
P-LF 100-8
E-LF 7.5m-1
E-LF 30m-8
E-LF 60m-2
E-LF 15m-1
Control for P-LF-8
Control for E-LF-6

## Slide 6
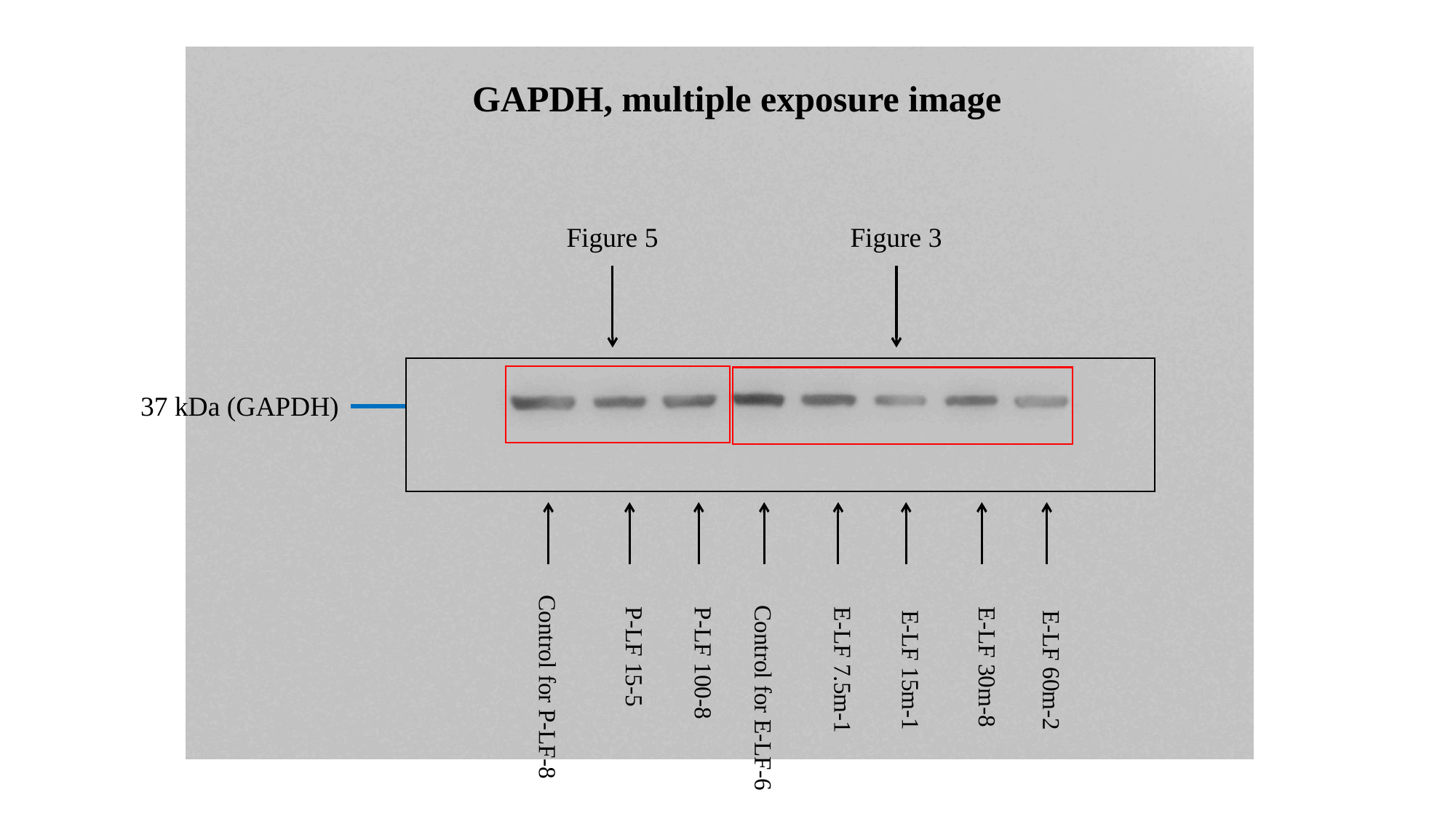

GAPDH, multiple exposure image
Figure 5
Figure 3
37 kDa (GAPDH)
P-LF 15-5
P-LF 100-8
E-LF 7.5m-1
E-LF 30m-8
E-LF 60m-2
E-LF 15m-1
Control for P-LF-8
Control for E-LF-6

## Slide 7
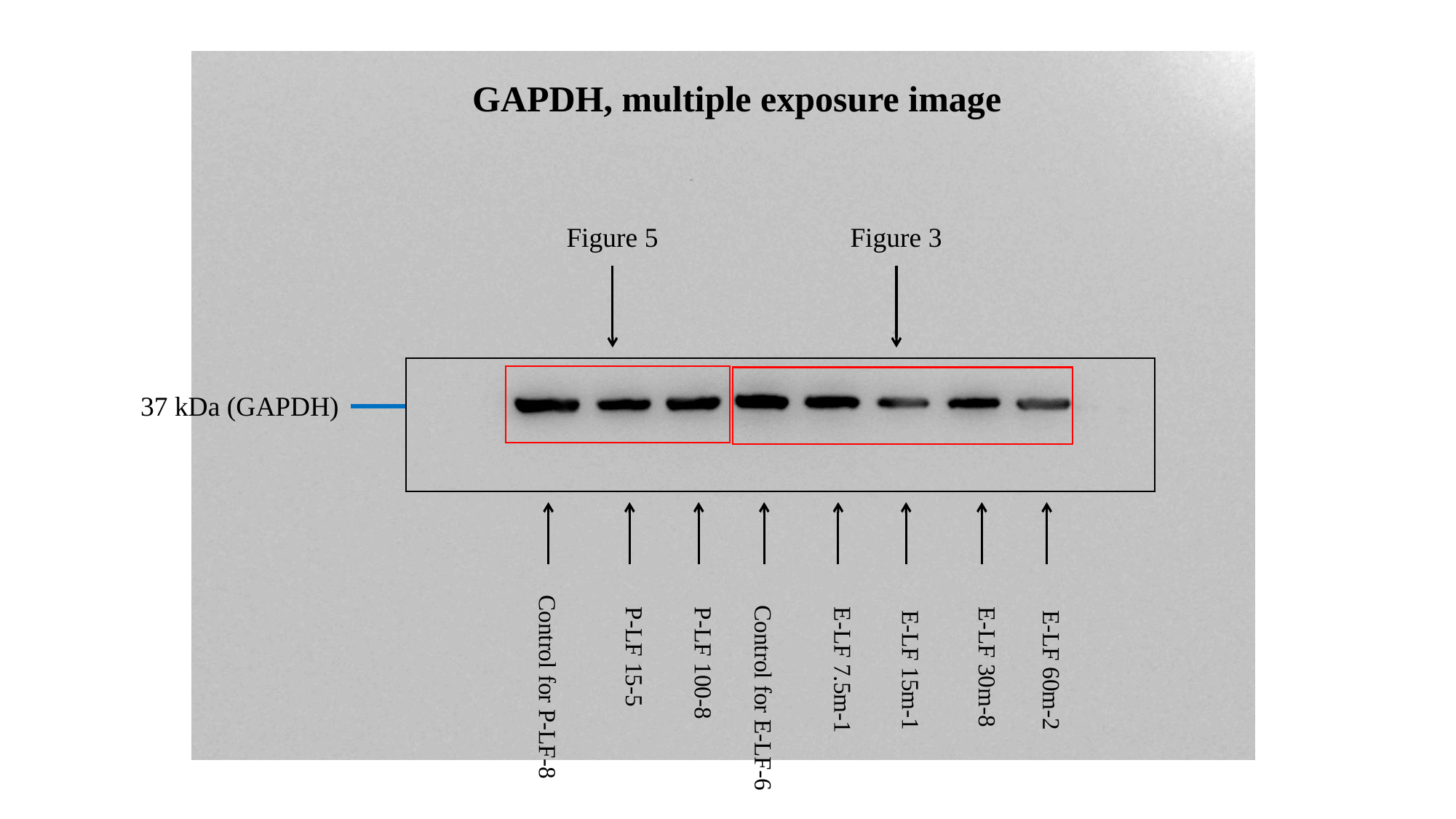

GAPDH, multiple exposure image
Figure 5
Figure 3
37 kDa (GAPDH)
P-LF 15-5
P-LF 100-8
E-LF 7.5m-1
E-LF 30m-8
E-LF 60m-2
E-LF 15m-1
Control for P-LF-8
Control for E-LF-6
